# Commentary: “Posttraining ablation of adult-generated olfactory granule cells degrades odor-reward memories”

**DOI:** 10.3389/fnins.2015.00110

**Published:** 2015-04-21

**Authors:** Joris Vangeneugden, Camille Mazo, Gabriel Lepousez

**Affiliations:** ^1^Department of Psychiatry and Neuropsychology, School for Mental Health and Neuroscience, European Graduate School of Neuroscience (EURON), Maastricht UniversityMaastricht, Netherlands; ^2^Laboratory for Perception and Memory, Department of Neurosciences, Institut PasteurParis, France; ^3^Unité Mixte de Recherche 3571, Centre National de la Recherche ScientifiqueParis, France

**Keywords:** adult neurogenesis, odor memory, olfactory granule cells, subcortical, neocortical storage, chemical ablation, olfactory association task

In the adult brain, hippocampus and olfactory bulb retain the remarkable capacity of generating new neurons and implementing them into existing neural circuitry. This phenomenon, adult neurogenesis, results from the presence of specific neurogenic zones, i.e., the sub-granular zone of the dentate gyrus and the sub-ventricular zone (SVZ). Within these regions, new immature neurons are continuously produced and then migrate out to their respective target circuits, differentiating either into glutamatergic neurons (dentate gyrus) or into mostly GABAergic interneurons called granule cells in the olfactory bulb. As a result, the production of new neurons throughout life is recognized as an extreme form of plasticity. The functional impact of recruiting newly-formed neurons on circuit functioning and their associated behaviors is a matter of intense investigation.

In the olfactory bulb, loss-of-function experiments have led to incongruent results regarding the role of newly-generated olfactory granule cells (OGC) in learning and memory. Previous methodologies to experimentally reduce neurogenesis consisted of administration of anti-mitotic or anti-proliferative drugs (1), focal irradiation (2) or use of genetic knockout mice (3). These studies have provided much insight, however are affected by (1) a-specificity leading to off-target effects such as targeting all proliferating cells within the hippocampus *and* the olfactory bulb with limited possibility to restrict this ablation in time, by (2) strong side neuro-inflammatory responses and by (3) brain-wide compensatory mechanisms, respectively.

To circumvent some of these issues, Arruda-Carvalho et al. (2014) investigated the functional significance of adult-born OGC by employing a powerful and selective “tag-and-ablate” genetic strategy while measuring the impact on behavior. To generate a conditional ablation of adult-born neurons, they crossed a line expressing a CRE-recombinase-dependent diphteria-toxine receptor (DTR) transgene with a strain carrying a tamoxifen-inducible CRE-recombinase cassette preferentially in SVZ Nestin-positive cells, that is to say preferentially in SVZ neural precursor cells compared to dentate gyrus precursor cells. In these double transgenic mice, short exposure to tamoxifen (TAM) triggers the expression of DTR in the neural stem cells and all their progeny. Following this tagging, a subsequent diphteria toxin injection specifically kills the tagged cells born after TAM injection, however the effectiveness of the ablation technique was not examined directly, via e.g. BrdU/NeuN or BrdU/Dcx staining. It is noteworthy that the administration of TAM induces a permanent recombination in neural stem cells. Thus, the tagged population 21 days following TAM injection included both 21 days-old OGC as well as younger immature neurons born during these last 21 days. Therefore, this methodology cannot reach the time selectivity of optogenetic approaches allowing precise manipulation of a restricted age-match cohort of adult-born neurons (Alonso et al., 2012; Gu et al., 2012).

Previously the authors used this technique to demonstrate that in the hippocampus contextual and spatial memories can be affected while similar ablation before learning had no effect (Arruda-Carvalho et al., 2011). Moreover, they further showed that these cells exert their influence in a maturation-dependent manner (Gu et al., 2012). Employing this “tag-and-ablate” strategy in the olfactory bulb the authors manipulate the duration between tagging and training and the duration between training and ablating in both adult and juvenile double transgenic mice. To discard the possible side effect of hippocampal neurogenesis ablation, the authors tested the effect of reducing adult-generated OGCs on a previously established hippocampal-independent associative olfactory task (Akers et al., 2011).

Odor-reward memories were clearly degraded after posttraining but not pre-training ablation of adult-born OGCs in both adult and young mice, indicating that memory storage and/or recall are affected by removal of adult-born neurons that had the opportunity to integrate in an odor-memory trace. On the other hand, the absence of effects after pre-training ablation suggests that adult-born OGCs are dispensable for acquisition of a new odor association and that existing mature OGCs provide a potential compensatory mechanism. Surprisingly, the temporal integration of these adult-born OGCs into existing memory traces turns out to be crucial. Degraded memories only occurred following ablation of synaptically integrated 21 days-old, but not immature 10 days-old neurons. Moreover, the ablation accounted only for more recent memory traces, i.e., occurring on behavioral testing 5 days but not 28 days after the pre-DT test.

This finding dovetails with a previous optogenetic study showing that reversibly silencing 4 weeks-old, but not 2 nor 8 weeks-old DGCs degraded performance in the water maze test (Gu et al., 2012). It furthermore suggests that, as in the hippocampus, a critical period in adult-born cells maturation exists during which they have a privileged role in memory storage and recall. Interestingly, during this critical period, adult-born OGCs exhibit heightened plasticity at input synapses from cortical regions (Lepousez et al., 2014). In addition, the preferred contribution of adult-born OGCs in recent memories is in line with a long-standing human neuropsychology tradition claiming a role for the neocortex in long-term storage of memory traces and the importance of memory transfer from sub-cortical to neocortical structures (Squire and Wixted, 2011; Herry and Johansen, 2014).

It remains to be determined whether adult-generated OGCs function as information-storing units incorporated into a broader circuit—possibly involving neocortical structures—or otherwise simply facilitate retrieval of information stored elsewhere (Arruda-Carvalho et al., 2014). Within the auditory system, associative fear memories can be supported by a merely neocortical microcircuit of disinhibition (Letzkus et al., 2011). During the acquisition phase, layer 1 interneurons activated by cholinergic basal forebrain afferents inhibit layer 2/3 parvalbumin interneurons resulting in disinhibition of pyramidal neurons. Optogenetic stimulation of this latter group of interneurons completely abolishes conditioned fear memory. It is tempting to think of an analogous circuitry subserving olfactory memory storage. A network involving preferential feedback cortical fibers to adult-born OGCs connectivity would support young odor memory while the circuit transfers to different units with time.

Recent insights into adult hippocampal (Sahay et al., 2011) and bulbar neurogenesis (Alonso et al., 2012) using gain-of-function approaches point toward the involvement of adult-born neurons specifically when discrimination tasks are challenging. In the case of posttraining OGCs ablation, the current study already demonstrates significant degradation of odor memories for a relatively easy task. It remains to be determined whether pre-training ablation would lead to learning impairment in the context of more difficult odor discriminations.

Granted the observation that spatial learning also depends on apoptosis of newborn hippocampal neurons (Dupret et al., 2007), it would be interesting to see how intricate compensatory mechanisms between survival and apoptosis play out in olfactory adult neurogenesis.

In summary, the authors suggest a first step toward a better understanding of the dynamics of memory formation such as acquisition, storage, consolidation, recall, and eventually transfer to other brain regions. Concerning the latter, the temporal relationship between neocortex and subcortical structures deserves thorough investigation.

## Conflict of interest statement

The authors declare that the research was conducted in the absence of any commercial or financial relationships that could be construed as a potential conflict of interest.
